# Supplementary material for: Factors associated with chronic kidney disease knowledge and preventive practices: An analytical cross-sectional study among patients with hypertension at Amana Regional Referral Hospital in Dar es Salaam, Tanzania
Source: PLoS One. 2025 Jan 8;20(1):e0317257. doi: 10.1371/journal.pone.0317257 (PMC11709296; doi:10.1371/journal.pone.0317257)
Supplement: Attachment — Submitted filename: Response to Reviewers.docx [file pone.0317257.s002.docx]

**Factors Associated with Chronic Kidney Disease Knowledge and Preventive Practices: An Analytical Cross-sectional Study among Patients with Hypertension at Amana Regional Referral Hospital in Dar es Salaam, Tanzania**

**Response to reviewer’s comments**

We would like to thank the reviewer for the comments provided to improve this study. We have revised the manuscript as shown in the point-to-point responses to reviewer’s comments provided in the table below.

perceiving symptoms as not serious, concerns about cost of care, responsibilities at home or work, and trouble finding or affording transportation

| **Comments** | **Authors Response** |
| --- | --- |
| **Journal requirements:** |  |
| Please ensure that your manuscript meets PLOS ONE's style requirements, including those for file naming. The PLOS ONE style templates can be found at https://journals.plos.org/plosone/s/file?id=wjVg/PLOSOne_formatting_sample_main_body.pdf and https://journals.plos.org/plosone/s/file?id=ba62/PLOSOne_formatting_sample_title_authors_affiliations.pdf | Thank you for the comment. We have read PLOS ONE’s style requirement and have used it to make revisions including file naming for our dataset |
| Thank you for stating the following financial disclosure: “This study was funded by the Ministry of Health in Tanzania”.  Please state what role the funders took in the study. If the funders had no role, please state: "The funders had no role in study design, data collection and analysis, decision to publish, or preparation of the manuscript." If this statement is not correct you must amend it as needed. Please include this amended Role of Funder statement in your cover letter; we will change the online submission form on your behalf. | Thank you for the comment. We have included the amended Role of Funder statement in our cover letter; we appreciate the assistance to change information in the online submission form on our behalf. |
| We note that your Data Availability Statement is currently as follows: [All relevant data are within the manuscript and its Supporting Information files.]  Please confirm at this time whether or not your submission contains all raw data required to replicate the results of your study. | We confirm at this time that our submission contains all raw data required to replicate the results of your study. We have uploaded data set used in our study as supporting information file. |
| **Additional Editor Comments:** |  |
| The authors could add information on the reliability of the Kiswahili version of the questionnaire if data is available from the pilot study. Reporting Cronbach’s alpha or any validation data would improve confidence in the tools used. | Thank you for the comment. We have added information on the reliability of the Kiswahili version by explaining the sample used to pilot the tool and how of the feedback from the pilot was useful in correcting and modifying the tool. In addition, we have reported the Cronbach’s alpha to explain the reliability of the tool used. |
| In the discussion, the authors could acknowledge potential confounding factors, such as access to healthcare and socioeconomic status, that may have influenced CKD knowledge and preventive practices. This would demonstrate an understanding of factors that could be addressed in future studies. | Thank you for the comment. We have acknowledged potential confounding factors such as access to healthcare and socioeconomic status as part of the discussion of limitations of the study. we have also modified the recommendations in the conclusion to accommodate this consideration |
| Reanalyzing the existing dataset could provide a more detailed interpretation of knowledge and preventive practices. Breaking down knowledge into specific domains (e.g., kidney function vs. CKD symptoms) could identify where the gaps in understanding are most prevalent. | Thank you for the comment. We have further analyzed the dataset to include knowledge assessment in specific CKD knowledge domains including the kidney functions, tests to assess kidney health, CKD risk factors, and signs and symptoms of CKD |
| The discussion section could be expanded to speculate on reasons for low adherence to preventive practices, such as regular renal checkups. The authors could reference existing literature on healthcare access or cultural factors in Tanzania that may influence health-seeking behavior. | Thank you for the comment. We have expanded reasons for low adherence to preventive practices, such as regular renal checkups. We have referenced existing literature on healthcare access and cultural factors in Tanzania that may have influenced health-seeking behavior. |
| The recommendations for educational programs could be strengthened by suggesting more targeted approaches, such as focusing on correcting specific misconceptions or emphasizing the importance of regular renal checkups, supported by references to similar interventions. | Thank you for the comment. We have strengthened the recommendations for educational programs by suggesting more targeted approached as commented |
| The study’s limitations, such as the cross-sectional design and inability to establish causality, should be explicitly discussed to enhance the integrity of the conclusions. | Thank you for the comment. We have further discussed the study’s limitations by explaining more on and inability of cross-sectional design to establish causality between knowledge and practice (although it is known that the two are associated as they influence each other), and potential confounding factors that were not assessed |
| If supplementary tables or additional analyses were conducted but not included, these could be added to provide further insights without the need for new data collection. | Thank you for the comment. We have performed further analysis to determine patients’ level of knowledge in specific CKD knowledge domains including the kidney functions, tests to assess kidney health, CKD risk factors, and signs and symptoms of CKD. This has also been included in the discussion. |
| **Reviewer #1:** |  |
| Ambikile et al present an interesting study that used patient questionnaires to assess factors associated with CKD knowledge and preventive practices among patients with hypertension in Dar es Salaam. The results revealed that >50% of study patients had high CKD knowledge and good CKD preventive practices. Importantly, higher CKD knowledge was associated with better preventive practices. The paper is well written with minor typos and grammatical errors. The paper is generalizable and the authors do a good job comparing their results to that of similar studies from other African and middle eastern countries. | We appreciate this comment |
| Could you elucidate more on the comorbidities that the study patients have? Ideally, this should be on table 1. The level of comorbidities may also influence patient knowledge and their motivation for CKD preventive measures. | Thank you for the comment. We agree comorbidities may have influenced patient knowledge and their motivation for CKD preventive measures. However, we did not assess comorbidities, therefore, we have included this as part of the study limitations in the discussion. |
| The study could be strengthened by showing the association between CKD knowledge and also blood pressure control or medication adherence. Do you have data on the blood pressure management of the patients in the study? | Thank you for the comment. Blood pressure control was one of the items assessed (Do you have regular hypertension screen in every week?) in DKC preventive practices. This was already shown in the results and included in the discussion. We did not look at the association between CKD knowledge and blood pressure control separately. Since 182 (98.9%) out of 184 patients had regular hypertension screen in every week, we have tried to run a Fisher’s Exact test to assess the association with CKD knowledge which has shown to be not significant. |
| **Reviewer #2:** |  |
| I have reviewed your submitted manuscript titled "Factors Associated with Chronic Kidney Disease Knowledge and Preventive Practices: An Analytical Cross-sectional Study Among Patients with Hypertension at Amana Regional Referral Hospital in Dar es Salaam, Tanzania" and have the following comments: | Thank you |
| Introduction: |  |
| Lines 72-73 - kindly reference the KDIGO sentence. | Thank you. Reference for the KDIGO sentence has been added |
| Methods: |  |
| Line 109 - be exact with the number of beds; it is a finite number and will be known. | Thank you. The exact bed capacity of the hospital is 362 and has been corrected |
| Line 141- Please clarify which cohort the excluded patients came from? Were they from the sample population i.e. the 184 patients or from the population of patients that had not met study eligibility ab initio (those who had chosen NO at simple randomization)? If from the sample population, could the patients who selected NO at random sampling not have been used as the pilot group? The exclusion of the data of the study sample population will reduce the effective sample size and the subsequent statistical analyses based on the downsized numbers. | We appreciate this concern. The patients who participated in the pilot stage that was carried out to test the data collection tool were the ones excluded from the analysis. Their number was calculated as 10% of the sample size but were not among 184 patients included in the study. Pretesting the data collection tool was done before the actual data collection was carried out. We have revised this part and provided more information to clarify the issue |
| Results: |  |
| Lines 291, 222 - the words "endorse", "endorsement" are conveying ambigous meanings with respect to the responses to the direct questions as itemized in Table 4. What do the authors mean by endorsement? Acknowledgment of the knowledge of the preventative measure or an ascertainment of the actual practice of preventative measures? Kindly find a word to express what is meant in these lines. | Thank you for this comment. We have addressed the word "endorse" to avoid ambiguous meaning by replacing it with the word “score” |
| Lines 219, 222 and Table 4 - the authors report that 68.4% of respondents engaged in 3 monthly renal checkups. Why were they doing this? Is this routine practice/standard of care in your hypertension clinic? | Thank you for the comment. This was an error. Tanzania, has adopted KDIGO and other international guidelines for monitoring kidney function among patients with hypertension. It is annually for well-controlled hypertension and no other risk factors, and every 3 to 6 months for patients with additional risk factors. We have corrected this error in the manuscript including Table 4. |
| Discussion: |  |
| The discussion needs to be more robust and not just a comparison of your findings with the findings of others. What are the implications of the similarities/differences of your results relative to others' results for your patient population, standard of care practices, possible policy establishment or change etc. For example, lines 250-251 can be discussed further as "about three-quarters were unaware that blood pressure levels can indicate kidney health." From a public health perspective, this is a very significant/concerning finding that should be discussed in an at-risk population in a world geographical region where preventative measures for CKD are to be emphasized and promoted. | Thank you for this comment. We have revised the discussion section to include more implications of findings. From a public health perspective, we have discussed further about three-quarters of patients who were unaware that blood pressure levels can indicate kidney health, considering at risk populations LMICs. |
| Limitations |  |
| Line 318 -319 - "the cross-sectional nature of the study prevents us from establishing a causal relationship between sociodemographic characteristics, CKD knowledge, and preventive practices." I do not believe that that there is a scientific need for causality in your study. The overarching relationship between knowledge and practice will be an association; knowledge or lack therefore influences practice but does not cause practice in a scientific sense. | We than you for the comment. We agree with this comment. However, based on the additional Editor’s comments, we have maintained this limitation and clarified the association between CKD knowledge and preventive practices as raised in the comment. |
| General comments: |  |
| A few grammatical errors through the manuscript; kindly have an independent person read through. The authors not likely to pick them due to familiarity with the text. | We thank you for the comment. We have obtained language editing assistance from an independent person who has corrected grammatical errors |
